# Supplementary material for: Soft robotic shell with active thermal display
Source: Sci Rep. 2021 Oct 8;11:20070. doi: 10.1038/s41598-021-99117-y (PMC8501096; doi:10.1038/s41598-021-99117-y)
Supplement: Supplementary file 1 — Supplementary Information 1. [file 41598_2021_99117_MOESM1_ESM.pdf]

Supplementary Materials for
Soft robotic shell with active thermal display

Yukiko Osawa,[1,4*†] Yuho Kinbara,[2] Masakazu Kageoka,[2] Kenji Iida,[3]
Abderrahmane Kheddar[1*†]

[1]CNRS-University of Montpellier, LIRMM, Montpellier, France
[2]Mitsui Chemicals, Inc., Tokyo, Japan
[3]Mitsui Chemicals Europe, Düsseldorf, Germany
[4]National Institute of Advanced Industrial Science and Technology (AIST), Japan

[*]These authors contributed equally to this work.
[†]Correspondence author, E-mail: yukiko.osawa-akiyama@aist.go.jp (Y.O.)

The PDF file includes:

Note S1. The water tank design.

Note S2. Theoretical model of the circulating water system.

Note S3. Temperature control system.

Note S4. Measurable temperature range.

Note S5. Peltier device's controllable temperature range and the time constant.

Note S6. Experimental setup.

Fig. S1. Example of physical human-robot interaction.

Fig. S2. Water tank responses.

Fig. S3. Thermal transfer model for the circulating water system.

Fig. S4. Temperature responses with various water flow velocities.

Fig. S5. Assessment for the heat transfer model.

Fig. S6. Overall control system.

Fig. S7. Temperature control of the Peltier device.

Fig. S8. Contacting the cover by a human hand.

Fig. S9. Assessment of temperature sensing system.

Table S1. The heating (cooling) time and the height of the water tank.

Table S2. Parameters of the circulating water model.

Table S3. Thermal parameters for the theoretical model.

Table S4. The water flow velocity and the convection heat-transfer coefficient.

Table S5. Identified parameter of the circulating water system.

Table S6. Parameters for experiments.

Table S7. Liquid’s heat conductivity.

Table S8. Performance comparison of the Pelter pumps.

Other Supplementary Material for this manuscript includes the following:

Movie S1. Thermal display of the robotic cover.

Movie S2. Capacitive sensing under controlling temperature (heating).

Movie S3. Capacitive sensing under controlling temperature (cooling).

Supplementary materials

Note S1. The water tank design

We designed and developed a customized water-tank for controlling the circulating water's temperature. Commercially available tanks are made of copper having high heat conductivity (386 W/mK [Holman(1990)]) but causes heat loss. Our experimental trials revealed that because of the channels built inside the copper tank, its capacity is not enough to heat and cool the water efficiently. Furthermore, the water inside the tank cannot be accessed to measure (see Fig. S2).

We investigated the water heating/cooling time to determine the height of the customized tank knowing that: Heating/cooling time in seconds = (amount of water in kg) $\times$ (end temperature in $^{\circ}$C $-$ start temperature in $^{\circ}$C) $\times$ (specific heat in joules/kg/$^{\circ}$C) / (heating(cooling) power in watts). The relationship between the tank's height and its heating (cooling) time is summarized in Table S1. We chose 6 mm for the height considering the response time, water volume, and making 4 mm holes to connect with the water pump by silicone pipe. Here, the cooling power was derived from the data sheet of the Peltier device (ETH-127-14-11-S).

Note S2. Theoretical model of the circulating water system

Heat transfer model for the whole system

The model parameters of the circulating water system are defined in Table S2. The circulating water system is subject to three thermal effects: absorbing/generating heat based on Peltier effect [Rowe(2018)], heat convection through the circulating water, and heat conduction through the surface layer (gel) of the robotic cover. These phenomena can be expressed by the thermal network method (see Fig. S3). Whole heat transfer is expressed as

$$C_w \frac{dT_w}{dt} = \frac{T_p - T_w}{R_w} - \frac{T_w - T_c}{R_c} \tag{1}$$

$$C_c \frac{dT_c}{dt} = \frac{T_w - T_c}{R_c} - \frac{T_c - T_a}{R_a}, \tag{2}$$

where T, C, R stand for heat flow, temperature, thermal capacitance, thermal resistance of the Peltier device (subscript p), circulating water (subscript w), the cover (subscript c), and an ambient element, respectively. Here, thermal capacitance and thermal resistance of the circulating water and the cover are derived as

$$C_w = \rho_w V_w c_{pw} \tag{3}$$

$$C_c = \rho_c V_c c_{pc} \tag{4}$$

$$R_w = \frac{1}{h_w A_w} \tag{5}$$

$$R_c = \frac{d_c}{\lambda_c A_c}, \tag{6}$$

where ρ, c_p, d, A, and V are the density, specific heat, thickness, surface area, and volume of the water (subscript w) and the cover (subscript c), respectively. The parameter λ_c and h_w denote the heat conductivity of the cover (gel layer) and the water's convection heat-transfer coefficient.

Heat convection model for the circulating water

The value of heat-transfer coefficient h switches between the cases where water is flowing or not; thermal phenomenon changes to pure heat conduction through the water when water flow stops. The Reynolds number (Re) is less than 2300 considering the water pump's capability in our system; the circulating water is a laminar flow. Using the empirical relationship for laminar flow and basic heat transfer model [Holman(1990), Cengel Yunus(2002)] heat transfer coefficient can be expressed as

$$\begin{cases} h = \dfrac{\lambda_w}{d_w} & (v = 0) \\ h = \dfrac{\lambda_w}{d_w}\left(3.66 + \dfrac{0.0668\,(d_w/L)\,\mathrm{RePr}}{1 + 0.04\,((d_w/L)\,\mathrm{RePr})^{2/3}}\right) & (v > 0) \end{cases} \tag{7}$$

derived from

$$h = \frac{\lambda_w}{d_w}\overline{\mathrm{Nu}}. \tag{8}$$

Here, the Reynolds number Re is defined as

$$\mathrm{Re} = \frac{\rho_w v d_w}{\mu}. \tag{9}$$

The parameters λ_w, d_w, L, v, μ, $\overline{\mathrm{Nu}}$, Pr stand for water's heat conductivity, density, total pipe length, water-flow velocity, viscosity, Nusselt number (average value), and Reynolds number, respectively. The equation (7) shows that the value of the convection heat-transfer coefficient significantly decreases when $v = 0$; it affects the time constant as follows.

Each layer's time constant

From the equations (3)-(6), the time constant of water and the cover are calculated as

$$R_w C_w = \frac{\rho_w V_w c_{pw}}{h A_w} \tag{10}$$

$$R_c C_c = \frac{\rho_c V_c c_{pc}}{\lambda_c A_c / d_c}. \tag{11}$$

In the case of the maximum water-flow velocity, the theoretical time constant of our experimental setup is 32 seconds (water) and 5 seconds (cover), respectively (37 seconds in total); the calculated values are rounded off from the theoretical values in Table S3. In reality, the total time constant takes longer, considering Peltier's time constant and heat loss effect. The relationship between the water velocity and the time constant is shown in Fig. S4 and Table S4.

Note S3. Temperature control system

Simplified model for controller

The heat transfer model can be simplified with a first-order lag system with a time delay as

$$T_c(s) = \frac{1}{1 + R_{\mathrm{com}} C_{\mathrm{com}} s} \exp(-L_d s) \left(T_p(s) - q_a\right), \tag{12}$$

where T_p, T_c, C_{com}, R_{com}, q_a, L_d, and s stand for temperature of the Peltier device and the cover, combined thermal capacitance, combined thermal resistance, heat loss, time delay, and the Laplace transform complex frequency, respectively. The heat loss term is expressed as

$$q_a = \frac{1}{\gamma_a} \left(T_c - T_a \right), \tag{13}$$

where T_a and γ_a denote ambient temperature and parameter related to the air thermal resistance, respectively. The experimental results are fitted with the model (see Fig. S5) and identified the parameters (see Table S5).

Temperature controller

The cover temperature was controlled by a model preview controller (MPC). The cost function with the constraints are calculated as

$$\min \sum_{i=1}^{H} \|\hat{T}_c(k+i|k) - T_c^{\text{cmd}}(k+i|k)\|_{W_1}^2 + \sum_{i=0}^{H-1} \|\hat{T}_p(k+i|k)\|_{W_2}^2 \tag{14}$$

subject to

$$T_c(k) = \frac{R_{\text{com}} C_{\text{com}}}{R_{\text{com}} C_{\text{com}} + t_s} T_c(k-1) + \frac{t_s}{R_{\text{com}} C_{\text{com}} + t_s} T_p(k-1-L_d/t_s) \tag{15}$$

$$T_{\text{min}}^{\text{th}} \leq T_p(k) \leq T_{\text{max}}^{\text{th}}, \tag{16}$$

where k, H, W_1, W_2, t_s, $T_{\text{min}}^{\text{th}}$, and $T_{\text{max}}^{\text{th}}$ stand for discrete time, prediction horizon, weight values of the cost function, sampling time, and minimum and maximum value of threshold of the calculated input, respectively. The first term of (14) is to make the temperature of the cover T_c follow its temperature command T_c^{cmd}, and the second one is for suppressing rapid change of the Peltier device's command. The discrete model in (15) was derived from (12) by discretizing based on the backward Euler method. Here, time delay L_d is a positive integer. Based on the command generated from MPC, the Peltier device's temperature was regulated by the proportional-integral (PI) controller. The constraint was decided considering safety ($T_{\text{max}}^{\text{th}}$ is

less than 77 °C) and the limitation of the amplifier ($T_{\rm min}^{\rm th}$ is more than 12 °C). The whole control system and the experimental parameters are shown in Fig. S6 and Table S6, respectively.

Note S4. Measurable temperature range

In our experimental setup, we use T-type thermocouples to measure temperature. The measurable temperature range is suitable for our applications (-252.87 °C to about 300 °C), knowing a stable and high accuracy temperature sensor [Powell(1974)]. The temperature obtained from the thermocouples is amplified between 0 and 5 voltage by the sensor amplifier (THAB-T-200); the measurable range is from -50 °C to 200 °C (the accuracy: ±0.625 °C), of which resolution is 0.02V/°C. The analog signals (voltage) are converted by a 16-bit analog-to-digital (AD) converter, reduced its noise by a low-pass filter (LPF). The cut-off frequency of the LPF is set to 5 Hz, as a result of different trials. We confirmed the sensor outputs by putting the sensor into hot/cold water, comparing it with the wireless digital thermometer (RS PRO 1319A) (see Fig. S9).

Note S5. Peltier device's controllable temperature range and the time constant

Comparing the cooling power of three kinds of Peltier devices (see Table S8), we chose the most powerful one (ETH-127-14-11-S). The controllable temperature range using our experimental setup is between 5 °C (-17 °C from the room temperature 22 °C: the electric power limitation) and 94 °C (+72 °C from the room temperature 22 °C: limitation for safety).

The responses of temperature control with proportional integrator (PI) controller (P gain:0.3, I gain: 0.01) is shown in Fig. S7. The transient response speed fixed a certain value due to the electric power limitation; this is the maximum speed of the Peltier device's response in our experimental setup. From the figure, the time constant of the Peltier device itself is 1–4.5 seconds (heating) and 3–4 seconds (cooling), respectively.

Experimental setup

The circulating water system consists of the micro water pump (D200S, maximum flow rate: 80 ml/min., pressure: 11 psi), a 3D printed small water tank (see Fig. 3, two Peltier devices (ETH-127-14-11-S; maximum temperature difference: 72 °C, cooling capacity: 77.1 W, voltage: 15.7 V, current: 8.5 A, area: 40 cm $\times$ 40 cm), and thermocouples (T type, Exposed Junction Wire Thermocouple). The water supplying port are connected by T-joint connection with 1/8 inch screw port. Each part composing the system is connected by a silicone pipe, of which the inner diameter and whole length are 2.5 mm and 1.46 m, respectively. The control system consists of a regular PC with Linux OS and 1.0 msec sampling time, 16 bit AD/DA boards, sensor amplifier (THAB-T-200), and three Micro-Power Motor Amplifiers (TA115, 150 W continuous, 325 W peak).

The tank part (the pink part of Fig. 3**B** was printed by AnyCubic 3D Printing (Photon Mono X), using Colored UV Resin (color: black, main material: resin and photoinitiator, liquid density: 1.184 g/cm^3, solid density: 1.100 g/cm^3, hardness(D): 79.0).

Thermal images and movies were captured by the thermal imaging camera (FLIR One Pro LT iOS; precision: $\pm$3 °C or $\pm$5 $\%$, temperature resolution: 0.1 °C.)

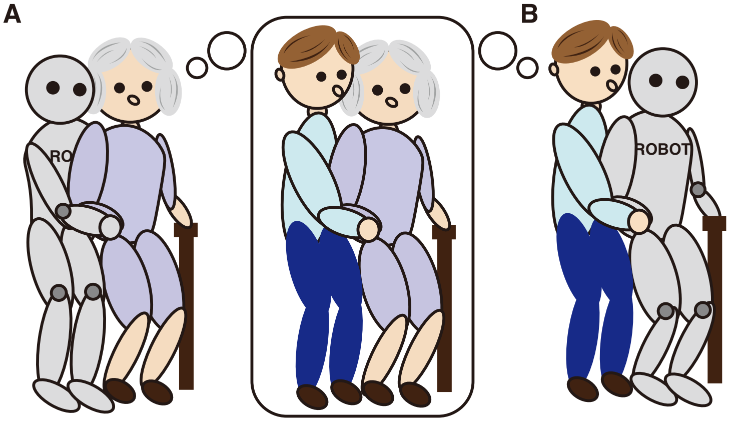

Fig. S1. Example of physical human-robot interaction. (**A**) Assisting motion for a frail person by means of service humanoid (e.g., site-to-stand motion in close human-humanoid multi-contact setting). (**B**) Emulating a frail person to train caregivers, here the humanoid plays the role of a controllable active manikin.

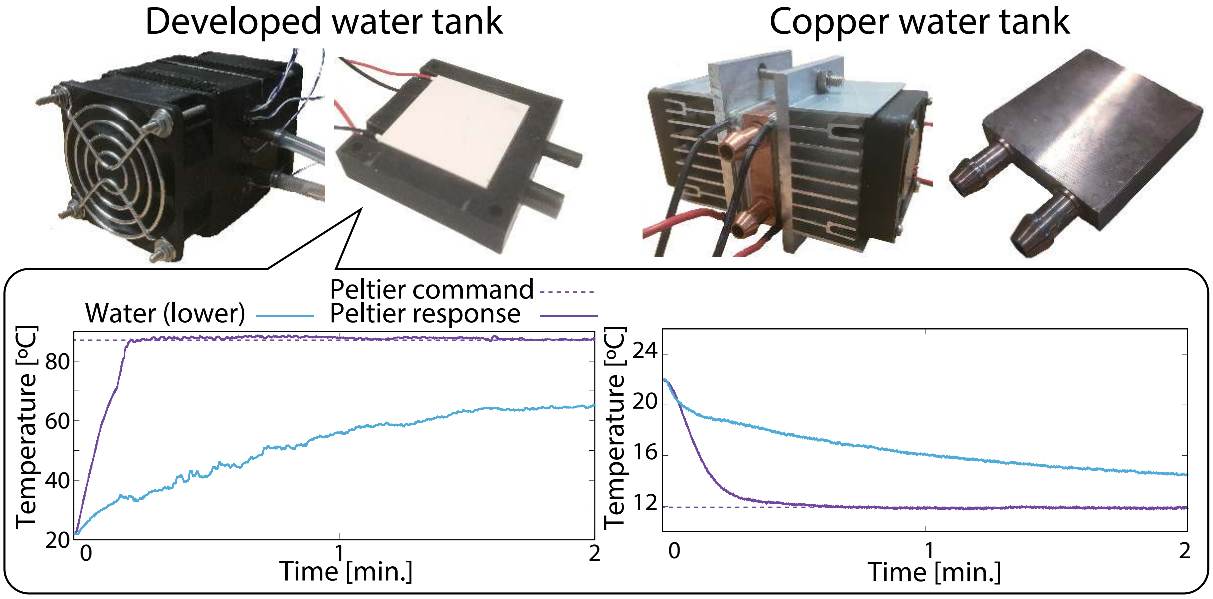

Fig. S2. Water tank response. Experimental results showing temperature responses of the water inside our customized water-tank in the case of heating and cooling. The Peltier device's desired temperature is set to +65 °C (heating) and -10 °C (cooling) from the room temperature (22 °C). Here, the tank's lower side sensor output (Fig. 3(**A**)) was plotted as a water temperature. For the commercially available copper tank, the water temperature inside the tank cannot be accessed directly; the thermocouples need to be attached to the copper tank wall. The customized tank can effectively heat and cool the water inside the tank, avoiding heat loss from the tank lateral sides.

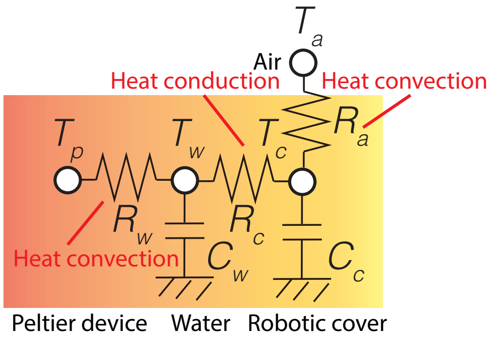

Fig. S3. Thermal transfer model for the circulating water system. The heat transfer model consists of two kinds of thermal effects: heat convection through the circulating water, and heat conduction through the surface layer of the cover. Additionally, heat radiation occurs from the cover surface (heat loss). These phenomena can be uniformly expressed by a thermal network method representing thermal properties as electric circuits analogies.

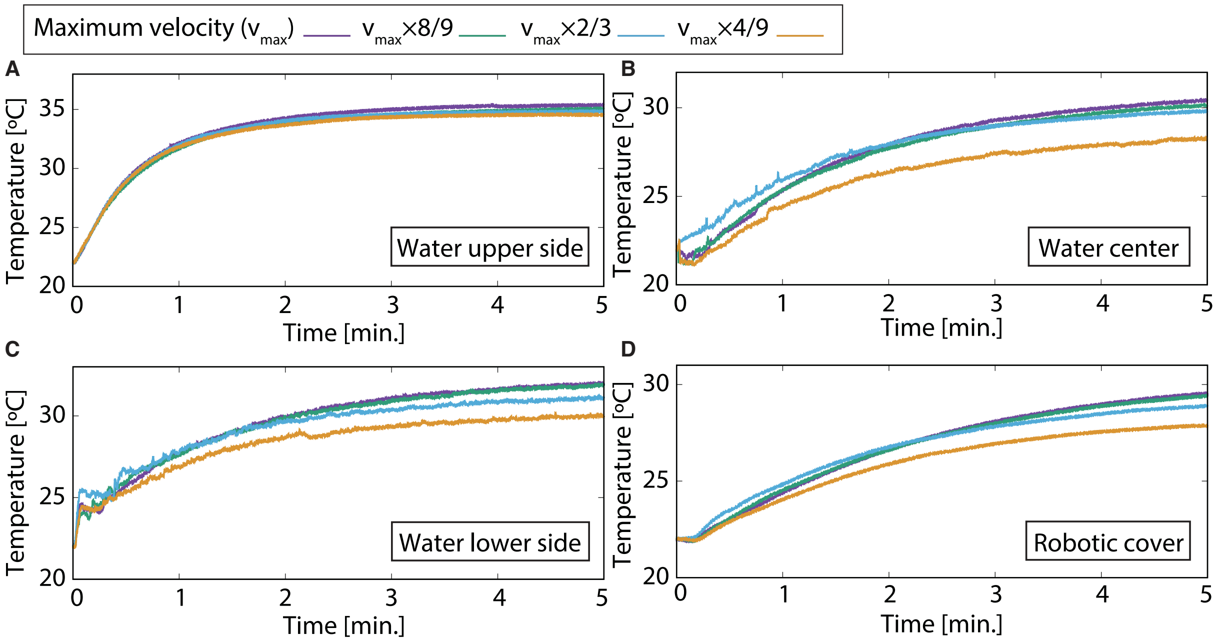

Fig. S4. Temperature responses with various water flow velocities. The temperature responses of water (upper, middle, lower side of the tank) and the cover surface were monitored in various velocity (maximum velocity $v_{\max}$, $v_{\max} \times 8/9$, $v_{\max} \times 2/3$, $v_{\max} \times 4/9$), while the Peltier device was controlled to 37 °C. As mentioned in Note S2 and Table S4, there is little deviations of the response within this velocity range; the case in $v_{\max} \times 4/9$ is too weak to circulate water smoothly, slowing down the response comparing with other cases.

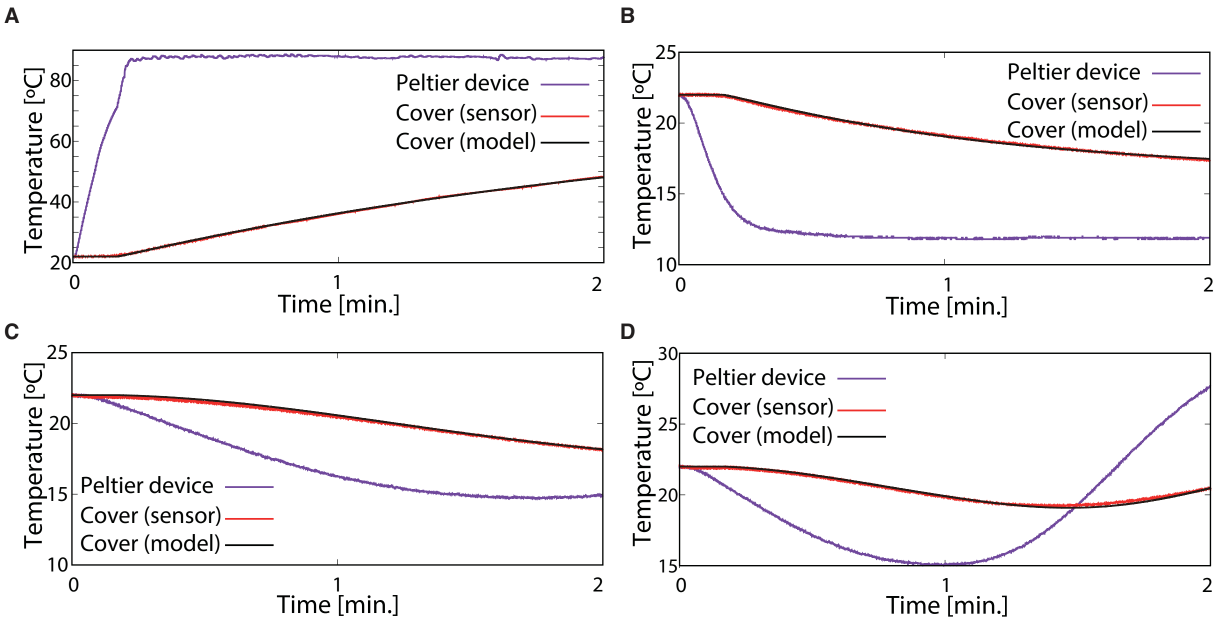

Fig. S5. Assessment for the heat transfer model. The measured temperature is compared to the estimated temperature derived from (12), circulating water with the water pump's maximum velocity. The identified parameters are summarized in Table S5. The Peltier devices is controlled to 87 °C (**A**) (heated), 12 °C (**B**) (cooled), 1/360 Hz sine wave (**C**), and 1/180 Hz sine wave (**D**). These results show that the temperature responses of the cover surface can be expressed as a time delay and a first-order lag system. Note that the identified parameters changed depending on the water temperature (fully heated (**A**) and cooled (**B**) cases, or the case kept at near room temperature (**C** and **D**). Thus, the appropriate parameters were used for the control system according to the temperature commands; the results in (**A**) are used for Fig. 6**A**, (**B**) are used for Fig. 6**B**, and (**C**) and (**D**) are used for Fig. 7.

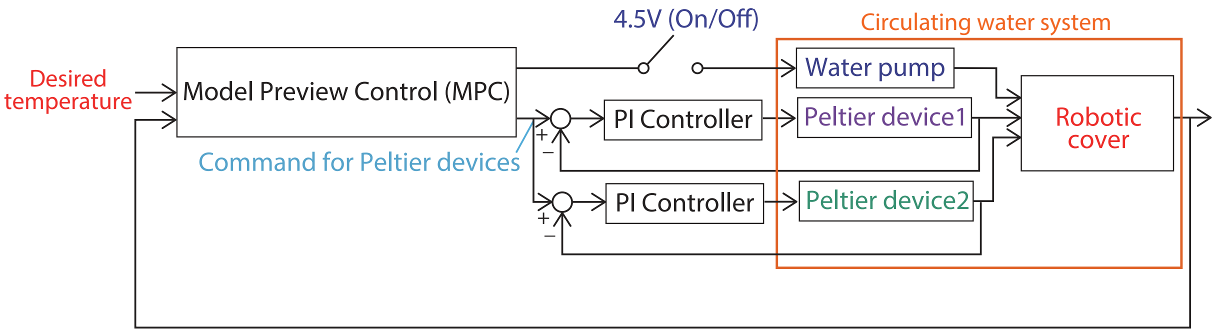

Fig. S6. Overall control system. Based on the intended temperature and the cover response, a model preview controller (MPC) generates the Peltier device's command (see Note S3). After reaching the cover response to its command, the water pump is turned off to keep the temperature; otherwise, it keeps the constant velocity of water flow.

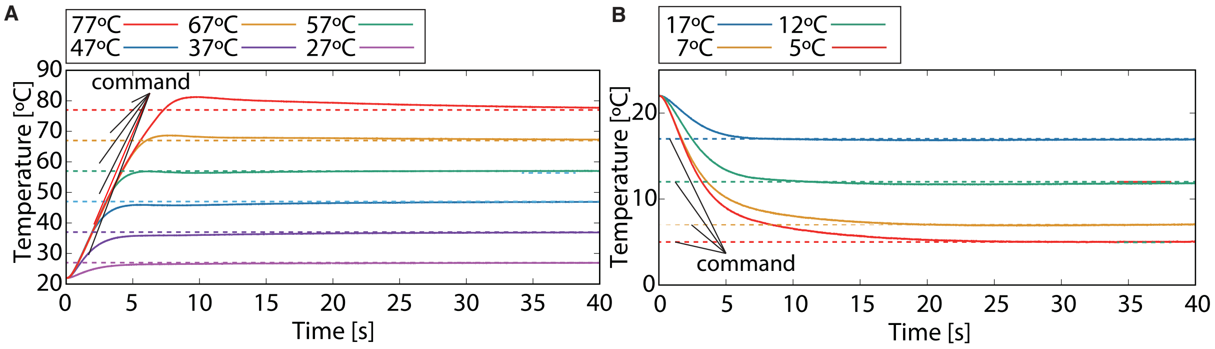

Fig. S7. Temperature control of the Peltier device. The Peltier device is controlled to 5 °C–77 °C (heated (**A**) and cooled (**B**)) with proportional integrator (PI) control (P gain:0.3, I gain: 0.01). The transient response speed fixed a certain value due to the electric power limitation; this is the maximum speed of the Peltier device's response in our experimental setup. The response speed depends on the desired and initial temperatures; e.g., it takes 3, 7, and 20 seconds to reach 27 °C, 77 °C, and 5 °C in our control system, respectively (room temperature: 22 °C).

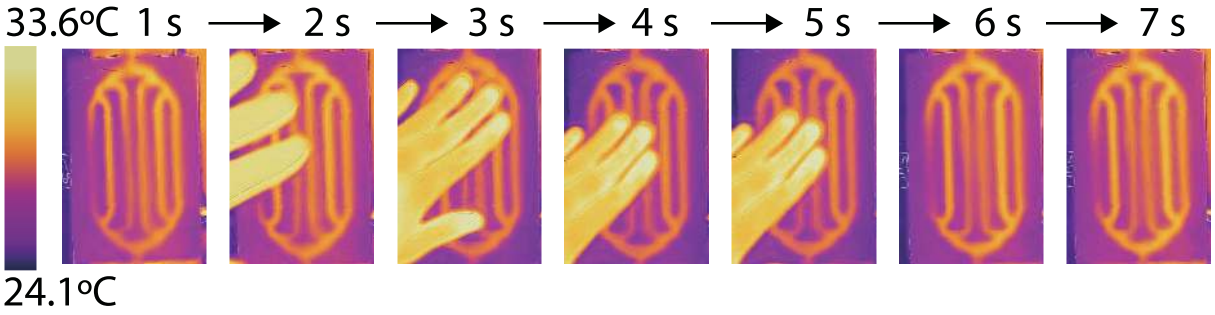

Fig. S8. Contacting the cover by a human hand. The cover is controlled to 30 °C and keeps the temperature while the human grabs it, giving a warm feeling to the person.

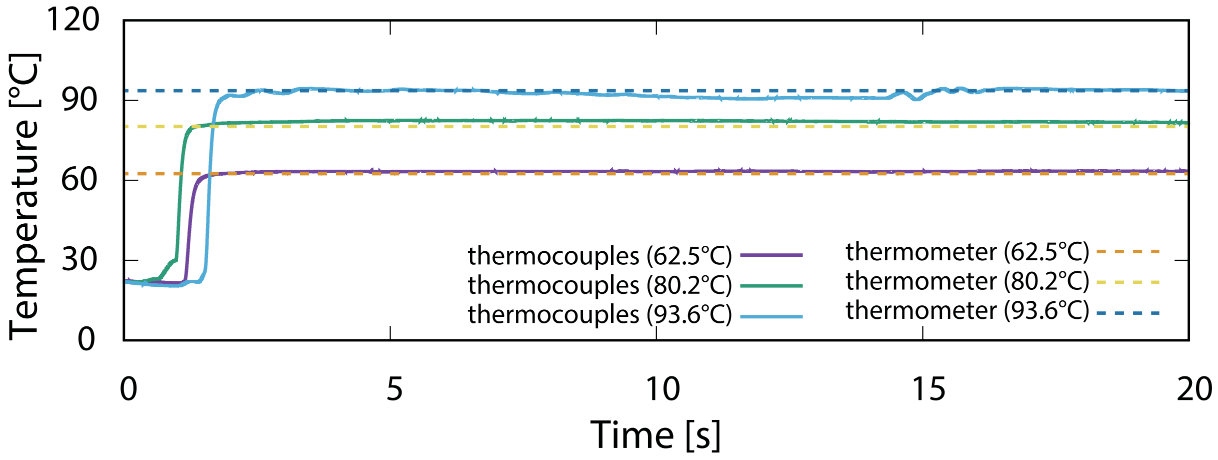

Fig. S9. Assessment of temperature sensing system. The experimental results show the outputs of thermocouples in hot water, compared with the wireless digital thermometer (RS PRO 1319A). These results suggest that the thermocouples can measure temperature correctly.

Table S1. The heating (cooling) time and the height of the water tank.

Height [m]	Cooling power [W]	Mass [kg]	Time [s]	
0.003	34	3.279 $\times 10^{-3}$	5.039	$\Delta T = 25$ (T_h=50 °C)
0.006	34	6.558 $\times 10^{-3}$	10.077	$\Delta T = 25$ (T_h=50 °C)
0.009	34	9.838 $\times 10^{-3}$	15.116	$\Delta T = 25$ (T_h=50 °C)
0.003	30	3.279 $\times 10^{-3}$	5.710	$\Delta T = 25$ (T_h=25 °C)
0.006	30	6.558 $\times 10^{-3}$	11.421	$\Delta T = 25$ (T_h=25 °C)
0.009	30	9.838 $\times 10^{-3}$	17.131	$\Delta T = 25$ (T_h=25 °C)

Table S2. Parameters of the circulating water model.

Parameter	Description
t	Time [s]
t_s	Sampling time [s]
T	Temperature [°C]
c_p	Specific heat of water [kJ·K/kg]
h	Convection heat-transfer coefficient $[\mathrm{W/m^2K}]$
λ	Thermal conductivity [W/mK]
v	Velocity of water flow [m/s]
ρ	Density [kg/m^3]
d	Diameter of the pipe [m]
L	Total pipe length [m]
A	Heat transfer surface area $[\mathrm{m}^2]$
V	Volume $[\mathrm{m}^3]$
μ	Viscosity [kg/ms]
R	Thermal resistance [K/W]
C	Thermal capacitance [J/K]
Nu	Nusselt number
Re	Reynolds number
Pr	Prandtl number
Subscript p	Peltier device
Subscript w	Water
Subscript c	Robotic cover
Subscript a	Ambient element
Subscript com	Combined value
$\hat{x}$	Estimated value of x

Table S3. Thermal parameters for the theoretical model.

Parameter				Theoretical value	Reference
R_w	A_w			3.2 $\times 10^{-3} \mathrm{m}^2$	Calculated (area of the Peltier devices)
	h	λ_w		0.614 $\mathrm{W/m} \cdot^\circ \mathrm{C}$	Theoretical value of water (26.67 °C) [Holman(1990)]
		d_w		2.5 $\times 10^{-3}$ m	Measured (circulating water system)
		L		1.46 m	Measured (circulating water system)
		Pr		5.85	Theoretical value of water (26.67 °C) [Holman(1990)]
		Re	ρ_w	995.8 $\mathrm{kg/m}^3$	Theoretical value of water (26.67 °C) [Holman(1990)]
			v	0.0679 $\mathrm{m/s}$	Calculated from pump's flow rate (average value)
			μ	8.6 $\times 10^{-4} \mathrm{kg/m} \cdot \mathrm{s}$	Theoretical value of water (26.67 °C) [Holman(1990)]
C_w	ρ_w			995.8 $\mathrm{kg/m}^3$	Theoretical value of water (26.67 °C) [Holman(1990)]
	c_p			4179 $\mathrm{J/kg} \cdot^\circ \mathrm{C}$	Theoretical value of water (26.67 °C) [Holman(1990)]
	V_{tank}			5.69 $\times 10^{-6}$ m^3	Measured using an injection
	V_w			1.29 $\times 10^{-5}$ m^3	Calculated ($V_{\text{tank}} + 2\pi \times d_w \times L$)
R_c	d_c			1.0 $\times 10^{-3}$ m	Measured (robotic cover)
	A_c			3.192 $\times 10^{-3}$ m^2	Calculated channel area (robotic cover)
	λ_c			0.37 $\mathrm{W/m} \cdot^\circ \mathrm{C}$	Measured by Mitsui Chemicals
C_c	ρ_c			1200 $\mathrm{kg/m}^3$	Measured by Mitsui Chemicals
	c_p			1720 $\mathrm{J/kg} \cdot^\circ \mathrm{C}$	Measured by Mitsui Chemicals
	V_c			3.192 $\times 10^{-6}$ m^3	Calculated ($A_c \times d_c$)

Table S4. The water flow velocity and the convection heat-transfer coefficient.
The convection heat-transfer coefficient h and the water's time constant are calculated by the formula mentioned in Note S2. (The water's thermal parameters are the theoretical values in 21.11 °C, and the length of water flow L is set to 1.46 m.) The maximum velocity (v_{max}) of the water pump is 0.0679 m/s. There is a big difference in the time constant between the water pump switches on and off. On the other hand, the time slightly changes when velocity is more than $v_{\text{max}} \times 1/3$. Therefore, the water pump switches off after the response reaches its commanded value, whereas the velocity keeps the maximum in our control system.

Velocity [m/s]	h [W/m$^2 \cdot$ °C]	Water's time constant [s]
0	201.333	120.732
$v_{\text{max}} \times 1/3$	744.674	32.642
$v_{\text{max}} \times 2/3$	752.221	32.314
v_{max}	759.588	32.001

Table S5. Identified parameter of the circulating water system.

Case	Time constant ($R_{\text{com}}C_{\text{com}}$)	Time delay (L_d)	γ_a in (13)
Fig. S5A (heating)	195 s	10.0 s	3.2
Fig. S5B (cooling)	115 s	10.0 s	1.2
Fig. S5C, D (sine wave)	96 s	10.0 s	8.2

Table S6. Parameters for experiments.

Parameter	Description	Value
t_s	sampling time	1.0 ms
$T^{\text{th}}_{\text{min}}$	Minimum value the constraint	12 °C
$T^{\text{th}}_{\text{max}}$	Maximum value the constraint	77 °C
H	Prediction horizon	15500
W_1	Weight value of eq. (14)	6.0
W_2	Weight value of eq. (14)	0.1
K_p	Proportional gain	0.3
K_i	Integral gain	0.01

Table S7. Liquid's heat conductivity.

Liquid	Heat conductivity [W/mK]	Reference
**Water**	**0.604**	[Holman(1990)] (21.11 °C)
Ammonia	0.521	[Holman(1990)] (20 °C)
Glycerin	0.289	[Holman(1990)] (20 °C)
Ethylen glycol	0.249	[Holman(1990)] (20 °C)
Sulfur dioxide	0.199	[Holman(1990)] (20 °C)
Engine oil	0.145	[Holman(1990)] (20 °C)
Dichlorodifluormethane (Freon-12)	0.073	[Holman(1990)] (20 °C)
Fluorinert electric liquid (FC-72)	0.057	[Holman(1990)] (20 °C)

Table S8. Performance comparison of the Peltier devices.

We compared three kinds of Peltier devices, of which driving power is less than the linear amplifier (Maximum power (peak): 162.5 W). Each cooling power in applying 4 A (maximum applicable current of our setup) is compared; the values are derived from the data sheet. T_h and ΔT stand for hot side temperature and temperature difference between the both sides of the Pelter pump, respectively. We chose the Peltier device (ETH-127-14-11-S) that is the most powerful among them.

Peltier device	ΔT= 25°C (T_h:50°C)	ΔT= 25°C (T_h:25°C)
ETH-127-14-11-S	34 W (4 A)	30 W (4 A)
	(datasheet description)	(datasheet description)
CP1.4-127-06L	32 W (4 A) (predicted)	26 W (4 A) (predicted)
(datasheet description)	29 W (3.6 A) – 34 W (4.8 A)	23.5 W (3.6 A) – 32 W (4.8 A)
TEC1-12706	29 W (4 A) (predicted)	26 W (4 A) (predicted)
(datasheet description)	18 W (3.0 A) – 43 W (4.5 A)	15 W (3.0 A) – 30 W (4.5 A)

References

[Cengel Yunus(2002)] A Cengel Yunus. Heat transfer: a practical approach, 2002.

[Holman(1990)] Jack P. Holman. *Heat transfer*, volume 1. McGraw-Hill, Inc, New York, 1990.

[Powell(1974)] Robert L Powell. *Thermocouple reference tables based on the IPTS-68*, volume 125. US National Bureau of Standards, 1974.

[Rowe(2018)] David Michael Rowe. *Thermoelectrics handbook: macro to nano*. CRC press, 2018.
